# Developing a predictive nomogram for colposcopists: a retrospective, multicenter study of cervical precancer identification in China

**DOI:** 10.1186/s12885-023-10646-3

**Published:** 2023-02-17

**Authors:** Peng Xue, Samuel Seery, Sumeng Wang, Yu Jiang, Youlin Qiao

**Affiliations:** 1 Department of Epidemiology and Biostatistics, School of Population Medicine and Public Health, Chinese Academy of Medical Sciences and Peking Union Medical College, 100730 Beijing, China; 2 Division of Health Research, Lancaster University, Lancaster, UK; 3 Department of Cancer Epidemiology, Chinese Academy of Medical Sciences and Peking Union Medical College, National Cancer Center, National Clinical Research Center for Cancer/Cancer Hospital, 100021 Beijing, China

**Keywords:** Predictive model, Colposcopy, Cervical precancer, Diagnosis

## Abstract

**Background:**

Colposcopic examination *with* biopsy is the standard procedure for referrals with abnormal cervical cancer screening results; however, the decision to biopsy is controvertible. Having a predictive model may help to improve high-grade squamous intraepithelial lesion or worse (HSIL+) predictions which could reduce unnecessary testing and protecting women from unnecessary harm.

**Methods:**

This retrospective multicenter study involved 5,854 patients identified through colposcopy databases. Cases were randomly assigned to a training set for development or to an internal validation set for performance assessment and comparability testing. Least Absolute Shrinkage and Selection Operator (LASSO) regression was used to reduce the number of candidate predictors and select statistically significant factors. Multivariable logistic regression was then used to establish a predictive model which generates risk scores for developing HSIL+. The predictive model is presented as a nomogram and was assessed for discriminability, and with calibration and decision curves. The model was externally validated with 472 consecutive patients and compared to 422 other patients from two additional hospitals.

**Results:**

The final predictive model included age, cytology results, human papillomavirus status, transformation zone types, colposcopic impressions, and size of lesion area. The model had good overall discrimination when predicting HSIL + risk, which was internally validated (Area Under the Curve [AUC] of 0.92 (95%CI 0.90–0.94)). External validation found an AUC of 0.91 (95%CI 0.88–0.94) across the consecutive sample, and 0.88 (95%CI 0.84–0.93) across the comparative sample. Calibration suggested good coherence between predicted and observed probabilities. Decision curve analysis also suggested this model would be clinically useful.

**Conclusion:**

We developed and validated a nomogram which incorporates multiple clinically relevant variables to better identify HSIL + cases during colposcopic examination. This model may help clinicians determining next steps and in particular, around the need to refer patients for colposcopy-guided biopsies.

**Supplementary Information:**

The online version contains supplementary material available at 10.1186/s12885-023-10646-3.

## Background

Approximately 600,000 women develop cervical cancer annually with a disproportionate burden of disease occurring in low- and middle-income countries (LMICs), where prevention programs are often limited [1]. With current population growth rates and demographic shifts such as aging, we can also expect this number to rise substantially [2]. The paradox here is that we can actually prevent this scenario from coming to pass, through screening and robust vaccination programs, yet many LMICs appear inhibited by their respective political economies. There are also more fundamental problems which need to be overcome in order to deliver effective cervical screening programs. These programs are necessarily multi-layered and involve cervical cytology testing e.g., Pap tests, as well as colposcopic assessments and biopsies. This means that effective cervical screening programs require experienced practitioners, who are not always readily available in LMICs [3]. Of course, new human papillomavirus (HPV) tests could prove useful in LMICs [4, 5]; however, processing all samples through cytology labs and byway of HPV-based screening actually appears to increase the number of false-negatives. This unacceptably high failure rate is likely to occur because cervical lesions related to HPV infections are probably milder and harder to identify than cytologic abnormalities [6, 7]. Therefore, we have to identify opportunities to improve screening and diagnostics, especially in cervical precancer identification.

In some ways, present colposcopic techniques lag behind HPV-related advances because the balance has rightly shifted toward prevention. At the other end of the diagnostic process, we have also witnessed the optimization of colposcopy-guided biopsy techniques. For example, the American Society of Colposcopy and Cervical Pathology (ASCCP) have proposed a low threshold biopsy protocol which involves multiple biopsies, targeting *all* areas. This low threshold protocol also includes acetowhite lesions and metaplasia, to improve high-grade squamous intraepithelial lesion or worse (HSIL+) detection [8, 9]. However, while this protocol will increase the reliability of both positive and negative results, we must maintain caution because biopsies are not without harm. In fact, the entire process can be traumatic and can have a negative impact on one’s self-efficacy and sexuality [10]. Unfortunately, screening and the diagnostic process generally, can also prevent women participating in other screening programs. This means, we have to improve mid-stage processes i.e. colposcopy, to avoid the twin traps in the diagnostic decision-making process. That is, we have to ensure we are not overly reliant upon less sensitive, less specific screening methods while preventing overdiagnosis.

There is no doubt that the decision-making processes are complex and decisions on biopsy, even among experienced clinicians, can vary. This means that there are subjective components which influence the decision to biopsy. These components can be debated from a number of different perspectives [11] although clinicians generally decide whether to biopsy, based upon previous experiences. Studies of this suggest the decision to biopsy is often influenced by the severity of a patient’s referral results [12, 13]. With colposcopic impressions, it may therefore be possible to quantify HSIL + risk, which clinicians can then use to develop evidence-based diagnostic decisions. This potential improvement encouraged us to develop and validate a predictive model which intercalates multiple clinically relevant variables to enhance early HSIL + case identification. The goal, of course, is to construct a simple, reliable nomogram which provides a reliable, individualized estimate for HSIL + risk.

## Methods

### Study design and participants

This retrospective multicenter study was conducted with six different hospitals across mainland China. Anonymized digital records were obtained to develop and validate a predictive model using colposcopy databases from four municipal or provincial hospitals. In order to maximize generalizability, this nomogram was also tested with patients enrolled from two additional hospitals in China.

Patients were assigned to one of two external validation sets. Statistically significant risk factors were identified through regression analysis. They were then intercalated into a coherent model which was converted into a nomogram for clinical practitioners. All patients with clear indications of abnormal screening results i.e., cytology and HPV testing, underwent colposcopic examination. Colposcopists assessed transformation zone (TZ) types and provided colposcopic impressions (i.e. normal/benign, (low-grade squamous intraepithelial lesion) LSIL, and HSIL+) following the international colposcopic terminology for each referred patient [14]. All colposcopic abnormalities were biopsied, and endocervical curettage was performed, if necessary. Biopsy-confirmed cases were considered eligible for this study.

Demographics and medical histories, including age, gravidity, parity, and menopause status were also obtained using digital medical records. Patients with incomplete information were excluded. We also excluded patients with a history of cervical physical therapy, surgical operations, and pelvic radiotherapy.

The Lower Anogenital Squamous Terminology (LAST) system was implemented and includes normal, LSIL, HSIL, and invasive cancers. All histology slides from punch biopsies, excision specimens and/or endocervical curettage were reviewed by histologists in the respective local hospitals. Any disagreements were resolved by a panel of independent expert histologists. The worst grade of dysplasia present was considered the final diagnosis.

This study was approved by Institutional Review Boards (IRB) in each of the participating hospitals. Research was conducted in accordance with the Declaration of Helsinki although, the requirement for written informed consent was waived by the respective IRBs because data were anonymized and retrospectively analyzed.

### Potential predictive variables

All risks factors, considered potentially useful for predicting HSIL + development, were identified through key articles [8, 9, 13]. Additional factors recommended by senior clinicians were considered for inclusion in the predictive model. The following assessable predictors emerged as consistent indicators i.e., age, cytology results, HPV status, TZ types and colposcopic impressions.

Considering the nomogram was designed for general practice, we also intercalated gravidity, parity, menopause, cervix visibility, size of cervical lesion area into the prediction model after initial analysis. Classified cytology results were also included in more detail, including those negative for intraepithelial lesions or malignancy (NILM), atypical squamous cells of undetermined significance (ASC-US), LSIL, atypical squamous cells which did not exclude high grade squamous intraepithelial lesion (ASC-H), and HSIL+, and HPV status included HPV negative, non-16/18 HPV positive, and HPV16/18 positive. The coding of these variables has been provided in the supplementary materials as Table S1.

### Outcome definitions

HSIL+, including HSIL and invasive cancers, were defined according to the LAST system, which are the most widely accepted, current international guidelines [15, 16]. The outcome of the predictive model was based on patient risk for developing HSIL + upon colposcopic examination. We used HSIL + as a hypothetical biopsy threshold in order to provide an additional margin of safety against misclassification. Therefore, we did not further distinguish between cervical intraepithelial neoplasia grade 2 (CIN2) or CIN3 among HSIL cases, even though many CIN2 cases are destined to regress [17]. Diagnoses based on HSIL + were determined by local histologists. If histological diagnosis or grade were not determined during the review, the case was referred to senior histologists, who were blinded to all other potential predictors.

### Model development and validation

Model development and validation were performed (and reported) according to the Transparent Reporting of a Multivariable Prediction Model for Individual Prognosis or Diagnosis (TRIPOD) guidelines [18]. The complete dataset was sampled randomly according to the distribution of histological results (HSIL + as opposed to < HSIL). Samples were then assigned to a training set for model development or to an internal validation set to assess performance at an approximate ratio of 7:3. This step was implemented to ensure comparability between the datasets.

Model development and internal validation were conducted in three stages. First, 10 variables were entered into the selection process. Then, least absolute shrinkage and selection operator (LASSO) was applied to reduce the number of candidate predictors and to select the strongest predictors to construct the model [19]. This LASSO approach penalizes the absolute size of model coefficients according to the value of λ. With larger penalties, the estimates of weaker factors shrink toward zero therefore, only the strongest predictors were accepted into the model.

Predictors identified using LASSO regression were entered into a multivariate logistic regression model. Those which were consistently statistically significant were used to construct the final prediction model. Multivariable logistic regression was used to calculate regression coefficients, and predictive strength was quantified as odds ratios (OR) with 95% confidence intervals (CI).

The model was calibrated to assess performance during development, and throughout internal and external validation. We also calculated discriminabilty to determine which patients were likely to develop HSIL+. Decision curves were generated to determine whether this model has clinical usefulness. Model calibration was assessed graphically using calibration plots, which intercalates predicted plots versus observed results. Model discrimination was determined by generating area under the curves (AUC). Decision curve analysis was performed for clinical use with a higher net benefit indicating enhanced clinical usefulness.

### Model presentation

A nomogram is a graphical calculation device which provides a probability of a specific outcome, based on the overall effect of pertinent factors. Therefore, nomograms can potentially provide individualized, evidence-based, reliable, risk estimates. The validated prediction model which included statistically significant risk factors was presented as a regression equation which was then converted into a nomogram. Each predictor value in the nomogram was assigned a regression weight so that the total score is equivalent to a linear predictor. For this model, logistic transformation was applied to the linear predictor to produce probabilities for developing HSIL+.

### Statistical analysis

Sample size was based on available data because there was no standard way to calculate sample size in advance. There were 10 candidate predictors and over 28 events per variable. This was considered sufficient to develop a stable model. Histological diagnosis was taken as golden standard. Receiver operating characteristic (ROC) curves were created by plotting the true positive rate (i.e. sensitivity) against a false positive rate (1-specificity). AUC values were generated for further comparative analysis.

Diagnostic indexes including accuracy, sensitivity, specificity, positive predictive value (PPV), and negative predictive value (NPV) were calculated using 95% CIs with different cut-off values selected using the Clopper-Pearson method [20]. All statistical tests were two-sided and 0.05 was established as the threshold for statistical significance. Data analyses were conducted using Stata (version 15.0) and R (version 3.6.1).

## Results

### Basic characteristics of the study population

**Fig. 1:**
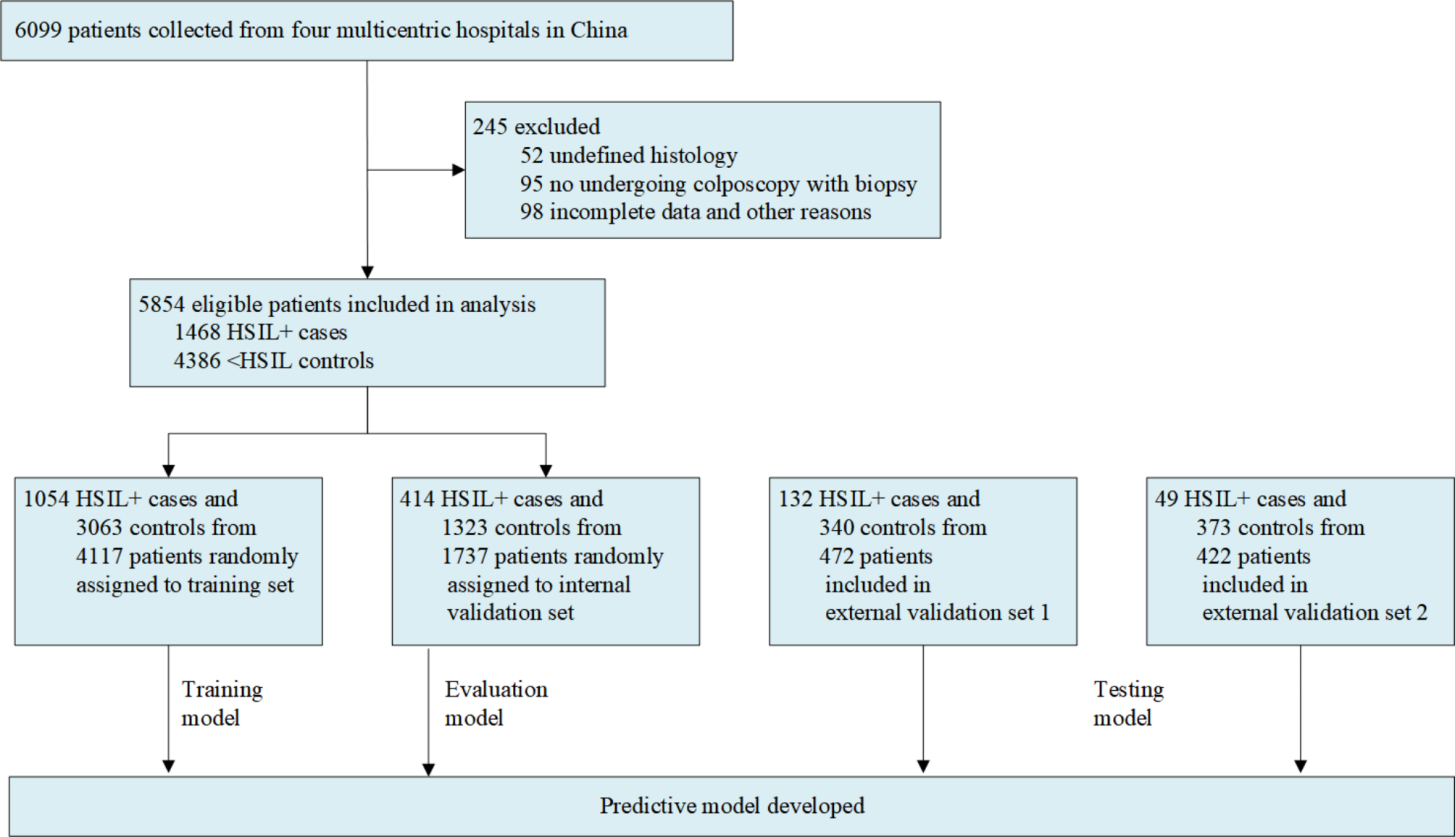
**Flowchart for the development and validation of predictive model**. (HSIL + cases included high-grade squamous intraepithelial lesion (HSIL) and invasive cancer. <HSIL controls included normal and low-grade squamous intraepithelial lesion (LSIL))

Throughout development and during internal validation, we identified 6,099 patients who had undergone colposcopic examination with biopsy in one of the four hospitals. Of these, 245 patients were excluded according to our predefined exclusion criteria. 5,854 patients were included, with 1,468 HSIL + cases and 4,386 < HSIL + controls. Please see Fig. 1 for a flowchart of patient selection and assignment.

Both external validation datasets were collected from April to November, 2021, to help ensure the generalizability of findings. 472 consecutive patients and 422 patients who had undergone colposcopy with biopsy were enrolled. The prevalence of HSIL + and the distribution of observed risk factors (except for those in menopause) were significantly different between the development dataset and the internal validation dataset. Sample characteristics are provided in Table 1.

**Table 1:** Demographics and clinical characteristics of study population

Characteristics No. (%)	Training set (n = 4117)	Internal validation set (n = 1737)	External validation set 1 (n = 472)	External validation set 2 (n = 422)
**Age (years)**				
< 30	769 (18.7)	306 (17.6)	60 (12.7)	47 (11.1)
30–39	1525 (37.1)	676 (38.9)	191 (40.5)	184 (43.6)
40–49	1096 (26.6)	440 (25.4)	151 (32.0)	140 (33.2)
50–59	578 (14.0)	238 (13.7)	59 (12.5)	40 (9.5)
> 59	149 (3.6)	77 (4.4)	11 (2.3)	11 (2.6)
**Gravidity**				
0	486 (11.8)	175 (10.1)	36 (7.6)	62 (14.7)
1–3	2863 (69.5)	1228 (70.7)	286 (60.6)	260 (61.6)
> 3	768 (18.7)	334 (19.2)	150 (31.8)	100 (23.7)
**Parity**				
0	733 (17.8)	292 (16.8)	64 (13.6)	97 (23.0)
1–2	3053 (74.2)	1309 (75.4)	336 (71.1)	314 (74.4)
> 2	311 (8.0)	136 (7.8)	72 (15.3)	11 (2.6)
**Menopause**				
No	3542 (86.0)	1456 (83.8)	408 (86.4)	365 (86.5)
Yes	575 (14.0)	281 (16.2)	64 (13.6)	57 (13.5)
**Cytology results**				
NILM	1722 (41.8)	688 (39.6)	219 (46.4)	272 (64.5)
ASC-US	1218 (29.6)	550 (31.7)	124 (26.3)	87 (20.6)
LSIL	688 (16.7)	290 (16.7)	69 (14.6)	28 (6.6)
ASC-H	153 (3.7)	83 (4.7)	21 (4.4)	25 (5.9)
HSIL	336 (8.2)	126 (7.3)	39 (8.3)	10 (2.4)
**HPV status**				
Negative	624 (15.2)	242 (13.9)	97 (20.6)	233 (55.2)
Non-16/18 hr-HPV	2084 (50.6)	878 (50.6)	245 (51.9)	140 (33.2)
HPV16/18	1409 (34.2)	617 (35.5)	130 (27.5)	49 (11.6)
**Cervix visibility**				
Inadequate	1304 (31.7)	550 (31.7)	220 (46.6)	226 (53.6)
Adequate	2813 (68.3)	1187 (68.3)	252 (53.4)	196 (46.4)
**TZ types**				
TZ1	1105 (26.8)	444 (25.6)	129 (27.3)	154 (36.5)
TZ2	1477 (35.9)	664 (38.2)	82 (17.4)	24 (5.7)
TZ3	1535 (37.3)	629 (36.2)	261 (55.3)	244 (57.8)
**Colposcopic impression**				
Normal/benign	1062 (25.8)	447 (25.7)	180 (38.1)	142 (33.6)
Low-grade	2110 (51.2)	913 (52.6)	177 (37.5)	241 (57.2)
High-grade	945 (23.0)	377 (21.7)	115 (24.4)	39 (9.2)
**Size of lesion area**				
< 1/3	1627 (39.5)	691 (39.8)	295 (62.5)	202 (47.9)
1/3 − 2/3	2214 (53.8)	923 (53.1)	161 (34.1)	187 (44.3)
> 2/3	276 (6.7)	123 (7.1)	16 (3.4)	33 (7.8)
**Histology**				
Normal/benign	1485 (36.1)	598 (34.5)	188 (39.8)	246 (58.3)
LSIL	1578 (38.3)	725 (41.7)	152 (32.2)	127 (30.1)
HSIL	1018 (24.7)	400 (23.0)	122 (25.9)	49 (11.6)
Cancer	36 (0.9)	14 (0.8)	10 (2.1)	None

Abbreviations: NILM, negative for intraepithelial lesion or malignancy; ASC-US, atypical squamous cells of undetermined significance; LSIL, low-grade squamous intraepithelial lesion; ASC-H, atypical squamous cells which did not exclude high grade squamous intraepithelial lesion; HSIL, high-grade squamous intraepithelial lesion; hr-HPV, high-risk human papillomavirus; TZ, transformation zone.

### Variable selection and model development

10 predictors measured in colposcopy clinics were included for LASSO regression modelling. Table S2 shows results from the candidate variables included in LASSO regression with corresponding coefficients for different values across the penalty parameter, λ. A lambda.min of 4.483 was observed, and all 10 predictor variables remained.

By increasing the λ value (to enhance shrinkage), we observed a lambda.1se of 32.262, with six predictors remaining. These were used to create the final model for validation. See Figure S1 for further details. The six remaining predictors were independent, statistically significant predictors for HSIL+, and were included in the logistic regression model, and for risk score calculations. The final model included age stratifications, cytologic results, HPV status, TZ types, colposcopic impression, and size of lesion area (see Table 2).

**Table 2:** Multivariate analysis of included predictors selected by LASSO regression procedure for detecting HSIL + in the development set

Included predictors	OR (95% CI)	*p* value
**Age (years)**		**0.022**
< 30	Reference	----
30–39	1.108 (0.815–1.507)	0.514
40–49	1.463 (1.064–2.011)	0.019
50–59	1.248 (0.860–1.811)	0.243
> 60	1.501 (0.807–2.795)	0.200
**Cytology results**		**< 0.001**
NILM	Reference	----
ASC-US	1.052 (0.809–1.368)	0.707
LSIL	1.525 (1.136–2.047)	0.005
ASC-H	2.225 (1.341–3.693)	0.002
HSIL	4.016 (2.708–5.955)	< 0.001
**HPV status**		**< 0.001**
HPV negative	Reference	----
Non-16/18 hr-HPV	2.028 (1.224–3.361)	0.006
HPV16/18 positive	5.002 (3.010–8.312)	< 0.001
**TZ types**		**< 0.001**
Type 1	Reference	----
Type 2	0.708 (0.548–0.916)	0.008
Type 3	0.490 (0.372–0.644)	< 0.001
**Colposcopic impression**		**< 0.001**
Normal/benign	Reference	----
Low-grade	3.252 (2.109–5.014)	< 0.001
High-grade	69.517 (44.269-109.165)	< 0.001
**Size of lesion area**		**0.001**
< 1/3	Reference	----
1/3 − 2/3	1.279 (1.001–1.635)	0.049
> 2/3	2.580 (1.698–3.918)	< 0.001

Abbreviations: LASSO, least absolute shrinkage and selection operator; HSIL+, high-grade squamous intraepithelial lesion or worse; OR, odds ratio; NILM, negative for intraepithelial lesion or malignancy; ASC-US, atypical squamous cells of undetermined significance; LSIL, low-grade squamous intraepithelial lesion; ASC-H, atypical squamous cells which did not exclude high grade squamous intraepithelial lesion; HSIL, high-grade squamous intraepithelial lesion; hr-HPV, high-risk human papillomavirus; TZ, transformation zone.

### Model performance: discrimination, calibration and decision curve analysis

**Fig. 2:**
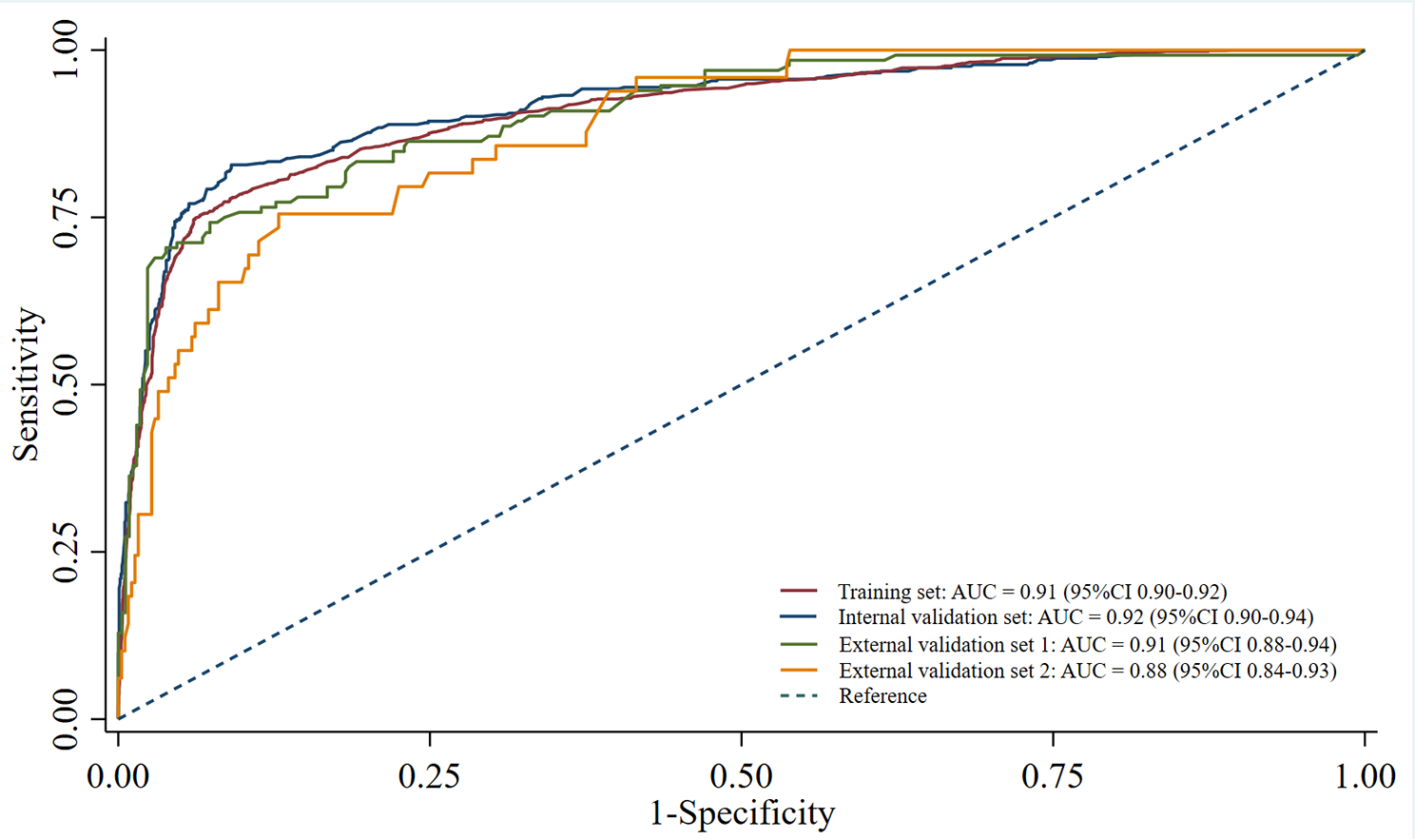
**Discrimination performance of predictive model in the training, internal and external validation sets**. (AUC = area under the curve)

The discriminating performance of the predictive model in training, and under internal and external validation can be seen in Fig. 2. The AUCs based on data from the training and internal validation sets were 0.91 (95%CI 0.90–0.92) and 0.92 (95%CI 0.90–0.94), respectively. The AUCs for HSIL + risk in external validation sets 1 and 2 were 0.91 (95%CI 0.88–0.94) and 0.88 (95%CI 0.84–0.93), respectively. Overall, AUC analysis indicated good model discriminability across all external datasets.

Table S3 shows the performance of indexes i.e., sensitivity, specificity, accuracy, PPV, and NPV, for each probability as a cut-off value in identifying individuals at risk of HSIL+. The resultant model prediction scores from 0 to 1, were categorized to balance sensitivity and specificity for detection of HSIL+. We also accepted a lower cut-off point of 0.10 with 92.4% sensitivity and 60.3% specificity for cases who did not require very frequent follow-ups. A higher cut-off point of 0.55 with 95.4% specificity and 70.4% sensitivity was selected for cases which required strict and timely follow-ups.

**Fig. 3:**
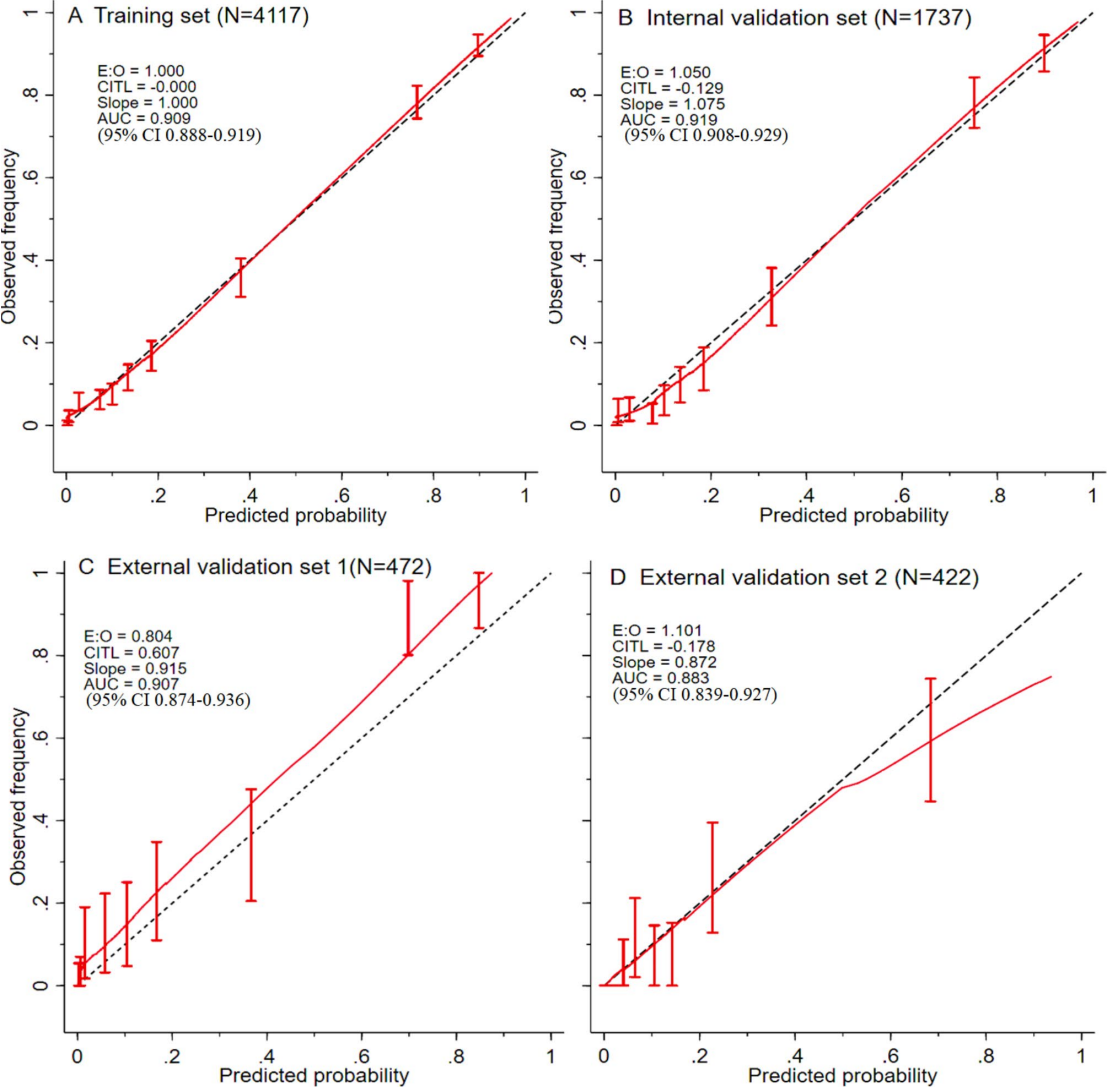
**Calibration plots of observed frequency and predicted probability for the predictive model in the training, internal and external validation sets**. (E:O, the observed divided by expected number, with a number close to 1 showing good model fit; CITL, calibration-in-the-large; AUC, area under the curve)

Figure 3 provides calibration plots for observed frequencies and predicted probabilities for the model in the training, internal and external validation sets. Overall calibrations (E:O, the observed divided by expected number) in each dataset were 1.00, 1.05, 0.80, and 1.10, respectively. AUCs based on data from both the training and internal validation sets were 0.91 (95%CI 0.89–0.92) and 0.92 (95%CI 0.91–0.93), respectively. Again, good model discrimination was observed based on comparable intercepts for predictive models under external validation sets with 0.91 (95%CI 0.87–0.94) and 0.88 (95% CI 0.84–0.93), respectively. This suggests the model is highly stable and has a low level of over-fitting.

**Fig. 4:**
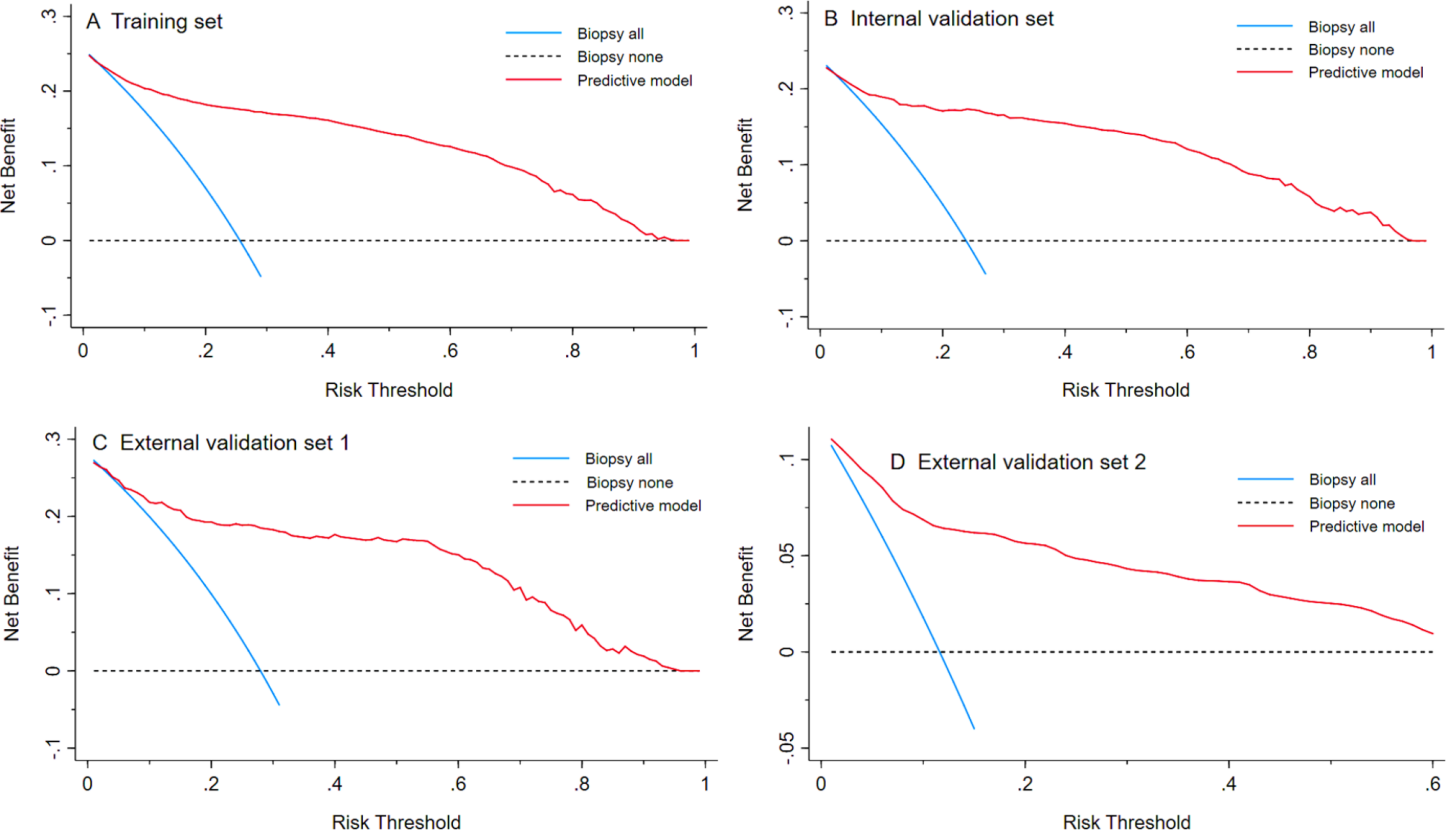
**Decision curve analysis depicting net benefit derived from the training, internal and external validation sets**. ((A) Training set; (B) Internal validation set; (C) External validation set 1; (D) External validation set 2. The horizontal and ordinate axis of this figure represented threshold probability, and the net benefit after the advantages were subtracted by the disadvantage, respectively. When a patient’s HSIL + risk reached a certain threshold, it was defined as high risk and biopsy measures were taken to further confirm final diagnosis. Decision curve analysis showed higher net benefit than biopsy for all patients, which suggests this model developed in this study is clinical usefulness)

Severity scores for predictive model were also analyzed with validation sets for a total of 24 invasive cancer cases. More complete data are provided in Table S4. Figure 4 provides decision curve analyses (DCA) for the training, internal and external validation sets. The horizontal and ordinate axis in this figure represents the threshold probability, and the net benefit after advantages were subtracted and counterbalanced according to disadvantages. When a patient’s risk of HSIL + reached a certain threshold, they were defined as high risk and biopsy measures were taken to confirm final diagnoses. Under training, and for internal and external validation sets, DCA found higher net benefits than biopsy for all patients, which indicates that the model developed is potentially clinically useful.

### Nomogram presentation

Figure S2 and Table S5 provide the weighted nomogram and risk prediction scores which generates an individual’s probability of developing HSIL+, at the colposcopy stage. Each predictor was assigned a specific grading value. When all six predictors were determined, the total number of points was calculated by adding all points together. The probability of developing HSIL + was then determined using a total point scale. Several practical colposcopy examples are provided in additional file.

## Discussion

In cervical cancer screening programs, colposcopic examination with biopsy has been widely accepted as a standard procedure for those with abnormal screening results. Colposcopy-guided biopsy decisions are crucial for deciding whether to follow-up, or to increase surveillance and for discussing further interventions. Taking multiple directed biopsies to improve HSIL + detection has also been widely accepted and applied in clinical practice [21, 22]. However, the decision to biopsy remains controversial among clinicians. In order to avoid missing HSIL + cases, clinicians may also prefer to biopsy, even in more unlikely instances. As has been mentioned, this can cause considerable psychological stress, which is in some instances totally unnecessary. There are also financial implications to excessive testing which increases the cost of cervical cancer screening programs [23]. Clearly, the one-size-fits-all approach is no longer appropriate. Therefore, we should make every effort to develop multivariable models which can individualize HSIL + risk estimates for colposcopists.

Here, we developed a novel predictive nomogram to identify patients who are likely to develop HSIL+. The idea was to develop and validate a model to be used with colposcopy to improve decisions around biopsy. Our model appears to have good discrimination and calibration when identifying those at high-risk of developing HSIL+. Findings were also internally and externally validated. These results were further supported by decision curve analysis, which showed a higher net benefit across almost the entire range of probability thresholds. The predictive model developed here included six statistically significant predictors, i.e., age, cytology results, HPV status, TZ types, colposcopic impression and size of lesion area. Each predictor used to calculate the risk of developing HSIL + are readily available to colposcopists, which adds to its applicability. If a patient’s estimated risk for developing HSIL + is low, clinicians may choose a wait-and-watch approach with follow-ups. For those with high-risk estimates, physicians should refer patients to colposcopic-guided biopsy for diagnosis; however, there may be opportunities to further develop this model.

More balanced cut-off values for detecting HSIL + might be selected to limit excessive biopsies although, there is a trade-off because sensitivity would likely drop. Another possibility for improving specificity while retaining high sensitivity might be to combine novel screening tests such as E6/E7mRNA, p16/Ki-67, or E6 oncoproteins. Two cut-off values have been suggested and depend upon the frequency of follow-up required. Given expert’s opinions and the findings from ROC curve analysis, we accepted a lower cut-off value of 0.10 with a 92.4% sensitivity and specificity of 60.3% for cases which did not require very frequent follow-ups. We also accepted a higher cut-off value of 0.55 with a 95.4% specificity and 70.4% sensitivity for those cases where strict and timely follow-ups were required. This would provide reassurance in negative results which can be shared with patients while reducing the number of unnecessary biopsies. Moreover, we should acknowledge that the predictive model commonly existed under-predicted risk in a population other than the population in which the model was developed. This is referred to as external validation sets. Variance in diagnostic performances in each external validation dataset may be attributed to the composition of the study sample, disease prevalence, clinical endpoints, and differences between colposcopic devices and/or approaches to biopsies. Each of these factors may cause bias which influences our ability to generalize model findings. Also, differences across populations highlights the need for more large-scale, multi-center training datasets, which could help to improve model generalizations and apply findings to different populations.

Clinical prediction models have been explored to predict cervical lesions, but few have been constructed for HSIL + predictions. Wu et al. [24] created and validated a logistic regression model for support vector machine (SVM) learning based on a multicenter cohort study of cervical cancer screening in China. Likewise, Karakitsos et al. [25] developed machine learning methods based on cytology, HPV status, E6/E7 mRNA test, and p16 immunostaining to build an algorithm to facilitate the classification of cervical intraepithelial neoplasia grade 2 or worse (CIN2+). Kahng et al. [26] developed an SVM model using age, cytology and presence of 15 HPV genotypes to identify the patient features that maximally contributed to progression to cervical precancers. Branca et al. [27] constructed comprehensive multivariate models by a panel of 13 biomarkers to predict CIN2+. However, these models have a number of different purposes and include different data sources, population characteristics, risk factors, and model performances varied, as did the extent of validation.

The aforementioned models were not developed for colposcopy, and included unattainable biomarkers such as E6 oncoprotein and p16/Ki-67, which are difficult to quantify in clinical practice. Recently, Li et al. [28] developed and validated a predictive model for endocervical curettage decision-making in cervical lesions based on colposcopic scenarios. Their prediction model intercalated screening results, TZ types, and colposcopic features which seem to be more feasible for colposcopic practice than previous studies suggest. However, their model has not been used for predicting cervical precancer risk during colposcopic examination. Other limitations of the previously published models were that they were not externally validated, and they developed models based on relatively small numbers of patients. This study was an attempt to fill this current knowledge gap by developing and externally validating a model which intercalates multiple predictors associated with colposcopy practice.

Previous studies [12, 29] have also found that combining these predictors could be used to identify patients who are likely to develop HSIL + for colposcopic biopsy. Although our study, is an attempt to determine risk at an earlier stage in the screening-diagnosis processes. We found the size of lesion area is an important predictor for detecting HSIL+. This is seemingly logical, yet it has not been described in the established guidelines, nor has it been factored into previous models. This means our model reduces the subjectivity involved in biopsy decision-making and is more appropriately evidence-based and provides a more reliable predictive tool. This study is the first to develop and validate a predictive model, which incorporates statistically significant variables as key predictors and thus advances our knowledge in the field.

Moving forward, the diagnostic accuracy of colposcopy *with* biopsy in the post-HPV vaccination era has become more unpredictable than ever. We must pay more attention to maintaining and optimizing the diagnostic accuracy of colposcopy with biopsy. Therefore, more scientific and technological research should be developed to improve diagnostic performances, especially in LMICs where there is a shortage of trained clinicians. In order to attain the WHO’s goal of eliminating cervical cancer worldwide by 2030 [30], we must adapt to advances and become early adopters of newly validated technologies. The findings from this study enable us to provide several recommendations for further research. Colposcopic practice needs to be further refined. Increasingly, evidence suggests that partial HPV genotyping types besides HPV16/18 carried a high risk of HSIL + that should be directly referred to colposcopy [31, 32].

We are witnessing an increasing number of innovations in colposcopic technologies. Novel colposcopy tools such as artificial intelligence (AI) guided digital colposcopy could help inexperienced clinicians standardize diagnostic procedures and to improve the accuracy of biopsy [33–35]. Although, these may not necessarily be readily available in LMICs and in areas where resources are unequally distributed. We are currently trying to build an interpretable cloud-based AI platform by combining our predictive model with a well-developed Colposcopic Artificial Intelligence Auxiliary Diagnostic System (CAIADS) from previous studies [36, 37]. It is hoped this will provide accessible telemedical assistance for LMICs. Although again, high-quality colposcopy training should be established to enhance cervical cancer diagnostics in LMICs. International colposcopy organizations should also provide continuous, updated, and mandatory accreditation to all colposcopy professionals, in order to provide the public with expertise and professionals with the support to fulfil their roles.

Predictors are readily available from digital clinical records in colposcopy clinics although, none have used LASSO regression analysis for predictor selection, and multicenter datasets for model development, and external validations to assess a model’s generalizability.. Therefore, this study had a number of advantages and undoubtedly adds to the evidence-base. One limitation of this study is that the model’s development was based on Chinese mainland women only, which may limit the generalizability to other populations. Other limitations include the computational complexity of the model and that our results were diagnosed by local senior clinicians. So, further research is needed to ensure this tool is easily applied and useful for less experienced clinicians. Finally, this study lacks a head-to-head comparison with clinicians due to the retrospective nature of this study design. The current model has the potential to help both patients and their providers although, further prospective clinical research would be useful to validate the effectiveness of model-assisted clinicians, generally.

## Conclusion

We developed and validated a prediction model by incorporating multiple clinically relevant variables to improve HSIL + case identification during colposcopic examination. This may help clinicians making decisions around colposcopy-guided biopsy procedures; however, further global, prospective research should be conducted before adopting this tool into clinical practice.

## Electronic supplementary material

Below is the link to the electronic supplementary material.

Supplementary Material 1

## Data Availability

Data available on request due to privacy/ethical restrictions. The data that support the findings of this study are available on request from the corresponding author. The data are not publicly available due to privacy or ethical restrictions.

## Abbreviations

LMIC: Low- and middle-income countries
LASSO: Least Absolute Shrinkage and Selection Operator
HSIL: High-grade squamous intraepithelial lesions or worse
LAST: Lower Anogenital Squamous Terminology
AUC: Area under the receiver operating characteristic curve
HPV: Human papillomavirus
ASCCP: American Society of Colposcopy and Cervical Pathology
LSIL: Low-grade squamous intraepithelial lesion
IRB: Institutional Review Boards
TZ: Transformation zone
CIN2: Cervical intraepithelial neoplasia grade 2
TRIPOD: Transparent Reporting of a Multivariable Prediction Model for Individual Prognosis or Diagnosis
OR: Odds ratios
CI: Confidence intervals
PPV: Positive predictive value
NPV: Negative predictive value
SVM: Support vector machine
AI: Artificial intelligence
CAIADS: Colposcopic Artificial Intelligence Auxiliary Diagnostic System
NILM: Negative for intraepithelial lesion or malignancy
ASC-US: Atypical squamous cells of undetermined significance
ASC-H: Atypical squamous cells which did not exclude high grade squamous intraepithelial lesion

## References

[1] Sung H, Ferlay J, Siegel RL, Laversanne M, Soerjomataram I, Jemal A, Bray F (2021). Global Cancer Statistics 2020: GLOBOCAN estimates of incidence and Mortality Worldwide for 36 cancers in 185 countries. CA Cancer J Clin.

[2] Bray F, Jemal A, Grey N, Ferlay J, Forman D (2012). Global cancer transitions according to the Human Development Index (2008–2030): a population-based study. Lancet Oncol.

[3] Xue P, Ng MTA, Qiao Y (2020). The challenges of colposcopy for cervical cancer screening in LMICs and solutions by artificial intelligence. BMC Med.

[4] Zhang J, Zhao Y, Dai Y, Dang L, Ma L, Yang C, Li Y, Kong L, Wei L, Zhang S (2021). Effectiveness of high-risk human papillomavirus testing for Cervical Cancer Screening in China: a Multicenter, Open-label, Randomized Clinical Trial. JAMA Oncol.

[5] Xue P, Gao LL, Yin J, Han LL, Zhao J, Li L, Seery S, Han XY, Li TY, Jiang Y (2019). A direct comparison of four high-risk human papillomavirus tests versus the cobas test: detecting CIN2 + in low-resource settings. J Med Virol.

[6] Leeson SC, Alibegashvili T, Arbyn M, Bergeron C, Carriero C, Mergui JL, Nieminen P, Prendiville W, Redman CW, Rieck GC (2014). The future role for colposcopy in Europe. J Low Genit Tract Dis.

[7] Lukic A, De Vincenzo R, Ciavattini A, Ricci C, Senatori R, Ruscito I, Frega A. Are We Facing a New Colposcopic Practice in the HPV Vaccination Era? Opportunities, Challenges, and New Perspectives. Vaccines (Basel)2021, 9(10). 10.3390/vaccines9101081 PMC8538171 34696189

[8] Wentzensen N, Schiffman M, Silver MI, Khan MJ, Perkins RB, Smith KM, Gage JC, Gold MA, Conageski C, Einstein MH (2017). ASCCP Colposcopy Standards: risk-based Colposcopy Practice. J Low Genit Tract Dis.

[9] Wentzensen N, Massad LS, Mayeaux EJ, Khan MJ, Waxman AG, Einstein MH, Conageski C, Schiffman MH, Gold MA, Apgar BS (2017). Evidence-based Consensus Recommendations for Colposcopy Practice for Cervical Cancer Prevention in the United States. J Low Genit Tract Dis.

[10] Khan MJ, Werner CL, Darragh TM, Guido RS, Mathews C, Moscicki AB, Mitchell MM, Schiffman M, Wentzensen N, Massad LS (2017). ASCCP Colposcopy Standards: role of Colposcopy, benefits, potential Harms, and terminology for colposcopic practice. J Low Genit Tract Dis.

[11] Schiffman M, Wentzensen N (2015). Issues in optimising and standardising the accuracy and utility of the colposcopic examination in the HPV era. Ecancermedicalscience.

[12] Silver MI, Andrews J, Cooper CK, Gage JC, Gold MA, Khan MJ, Massad LS, Parvu V, Perkins RB, Schiffman M (2018). Risk of cervical intraepithelial neoplasia 2 or worse by Cytology, Human Papillomavirus 16/18, and Colposcopy Impression: a systematic review and Meta-analysis. Obstet Gynecol.

[13] Ren H, Jia M, Zhao S, Li H, Fan S (2022). Factors correlated with the Accuracy of Colposcopy-Directed Biopsy: a systematic review and Meta-analysis. J Invest Surg.

[14] Bornstein J, Bentley J, Bösze P, Girardi F, Haefner H, Menton M, Perrotta M, Prendiville W, Russell P, Sideri M (2012). 2011 colposcopic terminology of the International Federation for Cervical Pathology and Colposcopy. Obstet Gynecol.

[15] Waxman AG, Chelmow D, Darragh TM, Lawson H, Moscicki AB (2012). Revised terminology for cervical histopathology and its implications for management of high-grade squamous intraepithelial lesions of the cervix. Obstet Gynecol.

[16] Perkins RB, Guido RS, Castle PE, Chelmow D, Einstein MH, Garcia F, Huh WK, Kim JJ, Moscicki AB, Nayar R (2020). : 2019 ASCCP risk-based Management Consensus Guidelines for abnormal cervical Cancer screening tests and Cancer Precursors. J Low Genit Tract Dis.

[17] Carreon JD, Sherman ME, Guillén D, Solomon D, Herrero R, Jerónimo J, Wacholder S, Rodríguez AC, Morales J, Hutchinson M (2007). CIN2 is a much less reproducible and less valid diagnosis than CIN3: results from a histological review of population-based cervical samples. Int J Gynecol Pathol.

[18] Moons KG, Altman DG, Reitsma JB, Ioannidis JP, Macaskill P, Steyerberg EW, Vickers AJ, Ransohoff DF, Collins GS (2015). Transparent reporting of a multivariable prediction model for individual prognosis or diagnosis (TRIPOD): explanation and elaboration. Ann Intern Med.

[19] Friedman J, Hastie T, Tibshirani R (2010). Regularization Paths for generalized Linear Models via Coordinate Descent. J Stat Softw.

[20] CLOPPER CJ, PEARSON ES: THE USE OF CONFIDENCE OR FIDUCIAL LIMITS ILLUSTRATED IN THE CASE OF THE BINOMIAL (1934). Biometrika.

[21] Wentzensen N, Walker JL, Gold MA, Smith KM, Zuna RE, Mathews C, Dunn ST, Zhang R, Moxley K, Bishop E (2015). Multiple biopsies and detection of cervical cancer precursors at colposcopy. J Clin Oncol.

[22] Bai A, Wang J, Li Q, Seery S, Xue P, Jiang Y (2022). Assessing colposcopic accuracy for high-grade squamous intraepithelial lesion detection: a retrospective, cohort study. BMC Womens Health.

[23] Wentzensen N, Walker J, Smith K, Gold MA, Zuna R, Massad LS, Liu A, Silver MI, Dunn ST, Schiffman M. A prospective study of risk-based colposcopy demonstrates improved detection of cervical precancers. *Am J Obstet Gynecol* 2018, 218(6):604.e601-604.e608. 10.1016/j.ajog.2018.02.009 PMC8771458 29462629

[24] Wu Z, Li T, Han Y, Jiang M, Yu Y, Xu H, Yu L, Cui J, Liu B, Chen F (2021). Development of models for cervical cancer screening: construction in a cross-sectional population and validation in two screening cohorts in China. BMC Med.

[25] Karakitsos P, Chrelias C, Pouliakis A, Koliopoulos G, Spathis A, Kyrgiou M, Meristoudis C, Chranioti A, Kottaridi C, Valasoulis G (2012). Identification of women for referral to colposcopy by neural networks: a preliminary study based on LBC and molecular biomarkers. J Biomed Biotechnol.

[26] Kahng J, Kim EH, Kim HG, Lee W (2015). Development of a cervical cancer progress prediction tool for human papillomavirus-positive Koreans: a support vector machine-based approach. J Int Med Res.

[27] Branca M, Ciotti M, Giorgi C, Santini D, Di Bonito L, Costa S, Benedetto A, Bonifacio D, Di Bonito P, Paba P (2008). Predicting high-risk human papillomavirus infection, progression of cervical intraepithelial neoplasia, and prognosis of cervical cancer with a panel of 13 biomarkers tested in multivariate modeling. Int J Gynecol Pathol.

[28] Li Y, Luo H, Zhang X, Chang J, Zhao Y, Li J, Li D, Wang W (2021). Development and validation of a clinical prediction model for endocervical curettage decision-making in cervical lesions. BMC Cancer.

[29] Del Pino M, Angeles MA, Martí C, Henere C, Munmany M, Marimon L, Saco A, Rakislova N, Ordi J, Torné A. Colposcopic Impression Has a Key Role in the Estimation of the Risk of HSIL/CIN3. Cancers (Basel)2021, 13(6). 10.3390/cancers13061224 PMC7999816 33799584

[30] WHO Director-. General calls for all countries to take action to help end the suffering caused by cervical cancer

[31] Stoler MH, Wright TC, Parvu V, Yanson K, Cooper CK, Andrews J (2019). Stratified risk of high-grade cervical disease using onclarity HPV extended genotyping in women, ≥ 25 years of age, with NILM cytology. Gynecol Oncol.

[32] Xue P, Seery S, Li Q, Jiang Y, Qiao Y. Risk-Based Colposcopy for Cervical Precancer Detection: A Cross-Sectional Multicenter Study in China. Diagnostics (Basel, Switzerland) 2022, 12(11). 10.3390/diagnostics12112585 PMC9689887 36359428

[33] Xue P, Wang J, Qin D, Yan H, Qu Y, Seery S, Jiang Y, Qiao Y (2022). Deep learning in image-based breast and cervical cancer detection: a systematic review and meta-analysis. NPJ Digit Med.

[34] Li Y, Chen J, Xue P, Tang C, Chang J, Chu C, Ma K, Li Q, Zheng Y, Qiao Y (2020). Computer-aided cervical Cancer diagnosis using time-lapsed colposcopic images. IEEE Trans Med Imaging.

[35] Li Y, Liu ZH, Xue P, Chen J, Ma K, Qian T, Zheng Y, Qiao YL (2021). GRAND: a large-scale dataset and benchmark for cervical intraepithelial neoplasia grading with fine-grained lesion description. Med Image Anal.

[36] Xue P, Tang C, Li Q, Li Y, Shen Y, Zhao Y, Chen J, Wu J, Li L, Wang W (2020). Development and validation of an artificial intelligence system for grading colposcopic impressions and guiding biopsies. BMC Med.

[37] Mendez MJG, Xue P, Qiao Y (2022). Cervical cancer elimination in the era of COVID-19: the potential role of Artificial Intelligence (AI)-guided digital colposcope cloud platform. EJGO.

