# Supplementary material for: Developing a predictive nomogram for colposcopists: a retrospective, multicenter study of cervical precancer identification in China
Source: BMC Cancer. 2023 Feb 17;23:163. doi: 10.1186/s12885-023-10646-3 (PMC9938572; doi:10.1186/s12885-023-10646-3)
Supplement: Supplementary file 1 — Supplementary Material 1 [file 12885_2023_10646_MOESM1_ESM.docx]

| **Table S1.** The coding of variables. | | |
| --- | --- | --- |
| Variable | Categorical/Binary | Coding |
| Age groups | Categorical | 1= <30  2= 30-39  3= 40-49  4= 50-59  5= >59 |
| Gravidity | Categorical | 1= 0  2= 1-3  3= >3 |
| Parity | Categorical | 1= 0  2= 1-2  3= >2 |
| Menopause (yes/no) | Binary | 0= No  1= Yes |
| Cytology results | Categorical | 1= NILM  2= ASC-US  3= LSIL  4= ASC-H  5= HSIL |
| HPV status | Categorical | 1= HPV negative  2= Non-16/18 HPV positive  3= HPV16/18 positive |
| Cervix visibility | Binary | 0=Adequate  1=Inadequate |
| TZ types | Categorical | 1= Type I  2= Type II  3= Type III |
| Colposcopic impression | Categorical | 1=Normal/benign  2=Low-grade  3=High-grade |
| Size of lesion area | Categorical | 1= <1/3  2= 1/3-2/3  3= >2/3 |
| Histology | Categorical | 1= Normal/benign  2=LSIL  3=HSIL  4=Cancer |

| **Table S2.** The candidate variables included in the LASSO regression and their corresponding coefficients for different values of the penalty parameter λ. | | |
| --- | --- | --- |
| Candidate variables | lambda. min= 4.483 | lambda.1se= 32.262 |
| (Intercept) | -4.1373200 | -3.0895047 |
| Age groups | | |
| <30 | Reference | Reference |
| 30-39 | 0.0399628 | ---- |
| 40-49 | 0.2822737 | 0.0637240 |
| 50-59 | 0.0842993 | ---- |
| >59 | ---- | ---- |
| Gravidity | | |
| 0 | Reference | Reference |
| 1-3 | 0.1830268 | ---- |
| >3 | 0.2104146 | ---- |
| Parity | | |
| 0 | Reference | Reference |
| 1-2 | ---- | ---- |
| >2 | 0.0933644 | ---- |
| Menopause | 0.2055230 | ---- |
| Cytology | | |
| NILM | Reference | Reference |
| ASC-US | ---- | ---- |
| LSIL | 0.3632808 | 0.1902227 |
| ASC-H | 0.6838122 | 0.3985344 |
| HSIL | 1.2808416 | 1.0439918 |
| HPV status |  |  |
| HPV negative | Reference | Reference |
| Non-16/18 HPV positive | 0.4720953 | ---- |
| HPV16/18 positive | 1.3712754 | 0.8137412 |
| Cervix visibility | -0.0797784 | ---- |
| TZ type | | |
| Type 1 | Reference | Reference |
| Type 2 | -0.2982939 | ---- |
| Type 3 | -0.7554805 | -0.2617581 |
| Colposcopic impression | | |
| Normal/benign | Reference | Reference |
| Low-grade | 1.1149975 | 0.5673140 |
| High-grade | 4.1772782 | 3.6520928 |
| Size of lesion area | | |
| <1/3 | Reference | Reference |
| 1/3-2/3 | 0.2182479 | 0.1341947 |
| >2/3 | 0.8920291 | 0.7150333 |

| **Tabel S3.** The performances of each probability as a cut-off value in identifying individuals at a high risk of HSIL+ | | | | | | |
| --- | --- | --- | --- | --- | --- | --- |
|  | Probability | Sensitivity, % (95%CI) | Specificity, % (95%CI) | Accuracy, % (95%CI) | PPV, % (95%CI) | NPV, % (95%CI) |
| 1 | 1.00 | 0 | 100 | 75.6(74.6-76.6) | 0 | 75.6(74.6-76.6) |
| 2 | 0.95 | 1.9 (1.2-2.6) | 100 | 76.0(75.0-77.0) | 100 | 75.9(74.9-76.9) |
| 3 | 0.90 | 16.4(14.6-18.2) | 99.8(99.7-99.9) | 79.4(78.4-80.4) | 95.7(93.3-98.1) | 78.7(77.7-79.7) |
| 4 | 0.85 | 30.8(28.6-33.0) | 99.1(98.8-99.4) | 82.4(81.5-83.3) | 92.0(89.7-94.3) | 81.6(80.6-82.6) |
| 5 | 0.80 | 43.7(41.3-46.1) | 98.3(97.9-98.7) | 85.0(84.1-85.9) | 89.3(87.2-91.4) | 84.4(83.5-85.3) |
| 6 | 0.75 | 53.9(51.5,56.3) | 97.5(97.1-97.9) | 86.8(86.0-87.6) | 87.3(85.3-89.3) | 86.7(85.8-87.6) |
| 7 | 0.70 | 60.1(57.7-62.5) | 96.7(96.2-97.2) | 87.8(87.0-88.6) | 85.6(83.6-87.6) | 88.2(87.4-89.0) |
| 8 | 0.65 | 64.9(62.6-67.2) | 96.3(95.8-96.8) | 88.6(87.8-89.4) | 84.9(82.9-86.9) | 89.5(88.7-90.3) |
| 9 | 0.60 | 68.1(65.9-80.3) | 95.8(95.2-96.4) | 89.0(88.3-89.7) | 84.0(82.0-86.0) | 90.3(89.5-91,1) |
| **10** | **0.55** | **70.4(68.2-72.6)** | **95.4(94.8-96.0)** | **89.3(88.5-90.0)** | **83.1(81.1-85.1)** | **90.9(90.1-91.7)** |
| 11 | 0.50 | 71.9(69.7-74.1) | 94.9(94.3-95.5) | 89.3(88.6-90.0) | 82.1(80.1-84.1) | 91.3(90.5-92.1) |
| 12 | 0.45 | 72.7(70.5-74.9) | 94.6(94.0-95.2) | 89.3(88.6-90.0) | 81.5(79.5-83.5) | 91.5(90.7-92.3) |
| 13 | 0.40 | 73.8(71.7-75.9) | 94.5(93.9-95.1) | 89.5(88.8-90.2) | 81.3(79.3-83.3) | 91.8(91.0-92.5) |
| 14 | 0.35 | 74.6(72.5-76.7) | 93.8(93.1-94.5) | 89.1(88.4-89.8) | 79.5(77.5-81.5) | 91.9(91.2-92.6) |
| 15 | 0.30 | 76.5(74.5-78.5) | 92.3(91.6-93.0) | 88.5(87.7-89.3) | 76.3(74.3-78.3) | 92.4(91.7-93.1) |
| 16 | 0.25 | 78.9(76.9-80.9) | 90.2(89.4-91.0) | 87.4(86.6-88.2) | 72.2(70.1-74.3) | 93.0(92.3-93.7) |
| 17 | 0.20 | 81.7(79.8-83.6) | 85.3(84.3-86.3) | 84.4(83.5-85.3) | 64.3(62.2-66.4) | 93.5(92.8-94.2) |
| 18 | 0.15 | 87.4(85.8-89.0) | 75.3(74.1-76.5) | 78.3(77.3-79.3) | 53.4(51.5-55.3) | 94.9(94.2-95.6) |
| **19** | **0.10** | **92.4(91.1-93.7)** | **60.3(59.0-61.6)** | **68.1(67.0-69.2)** | **42.9(41.3-44.5)** | **96.1(95.4-96.8)** |
| 20 | 0.05 | 96.7(95.8,97.6) | 40.0(38.7-41.3) | 53.9(52.7-55.1) | 34.3(32.9-35.7) | 97.4(96.7-98.1) |
| 21 | 0.00 | 100 | 0 | 24.4(23.4-25.4) | 24.4(23.4-25.4) | 0 |

| **Table S4.** Severity scores of predictive model evaluation from validation sets for cases of invasive cancer. | | | | | | |
| --- | --- | --- | --- | --- | --- | --- |
| Age (year) | Cytology  results | HPV status | TZ types | Coloscopic impression | Size of lesion area | Model severity score |
| 54 | NILM | HPV16/18 | 3 | High-grade | 1/3-/2/3 | 0.751 |
| 45 | HSIL | HPV16/18 | 3 | High-grade | <1/3 | 0.876 |
| 49 | NILM | HPV16/18 | 3 | High-grade | 1/3-/2/3 | 0.730 |
| 45 | NILM | HPV16/18 | 3 | High-grade | >2/3 | 0.786 |
| 38 | ASC-US | HPV16/18 | 2 | High-grade | <1/3 | 0.778 |
| 37 | HSIL | HPV16/18 | 3 | High-grade | >2/3 | 0.921 |
| 39 | NILM | HPV16/18 | 1 | High-grade | 1/3-/2/3 | 0.834 |
| 26 | HSIL | HPV16/18 | 3 | High-grade | >2/3 | 0.913 |
| 24 | ASC-US | Non-16/18 hrHPV | 3 | High-grade | 1/3-/2/3 | 0.554 |
| 28 | HSIL | HPV16/18 | 2 | High-grade | >2/3 | 0.938 |
| 33 | HSIL | Negative | 3 | High-grade | 1/3-/2/3 | 0.602 |
| 54 | HSIL | HPV16/18 | 3 | High-grade | 1/3-/2/3 | 0.915 |
| 62 | HSIL | Non-16/18 hrHPV | 3 | High-grade | 1/3-/2/3 | 0.835 |
| 38 | HSIL | HPV16/18 | 1 | High-grade | >2/3 | 0.961 |
| 56 | HSIL | Negative | 3 | High-grade | 1/3-/2/3 | 0.655 |
| 54 | NILM | HPV16/18 | 3 | High-grade | <1/3 | 0.689 |
| 40 | LSIL | HPV16/18 | 3 | High-grade | <1/3 | 0.789 |
| 47 | HSIL | HPV16/18 | 2 | High-grade | <1/3 | 0.910 |
| 41 | NILM | HPV16/18 | 2 | High-grade | <1/3 | 0.741 |
| 40 | HSIL | HPV16/18 | 1 | High-grade | <1/3 | 0.936 |
| 48 | ASC-US | HPV16/18 | 3 | High-grade | 1/3-/2/3 | 0.788 |
| 25 | HSIL | HPV16/18 | 2 | High-grade | <1/3 | 0.890 |
| 48 | HSIL | Non-16/18 hrHPV | 3 | High-grade | <1/3 | 0.747 |
| 61 | HSIL | Non-16/18 hrHPV | 3 | High-grade | <1/3 | 0.788 |

**Figure S1.** The progress of LASSO regression selecting the candidate variables and the penalty parameter λ.


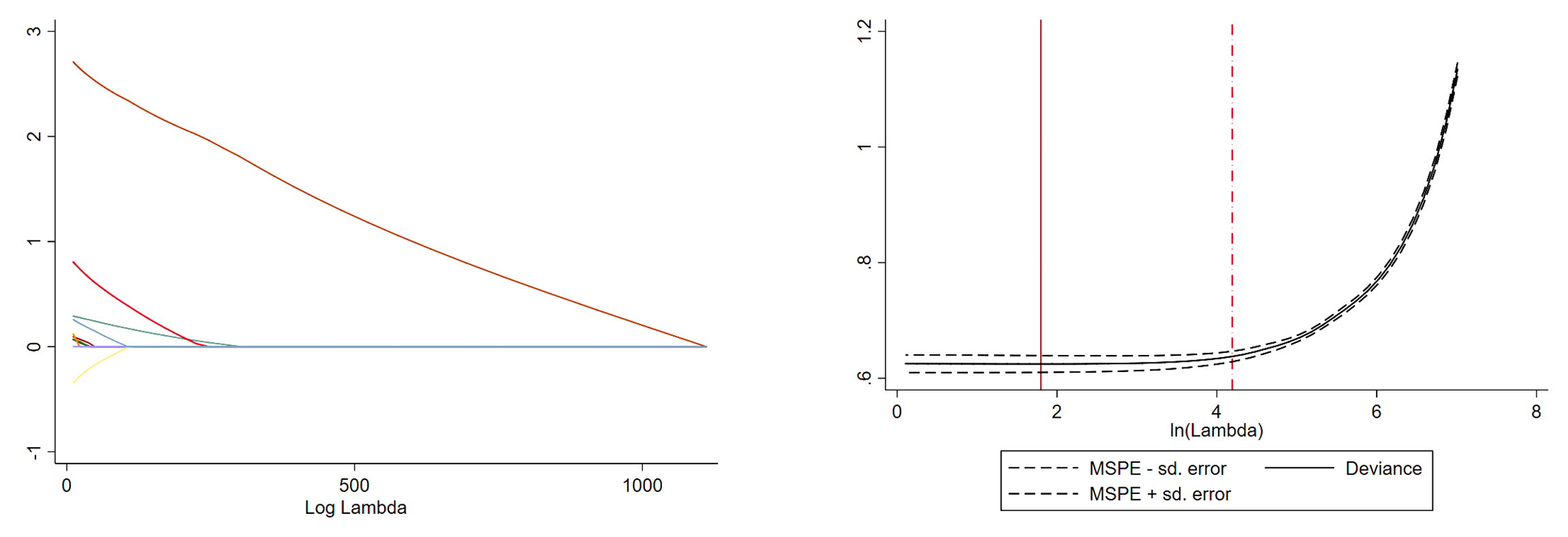
**A B**

**
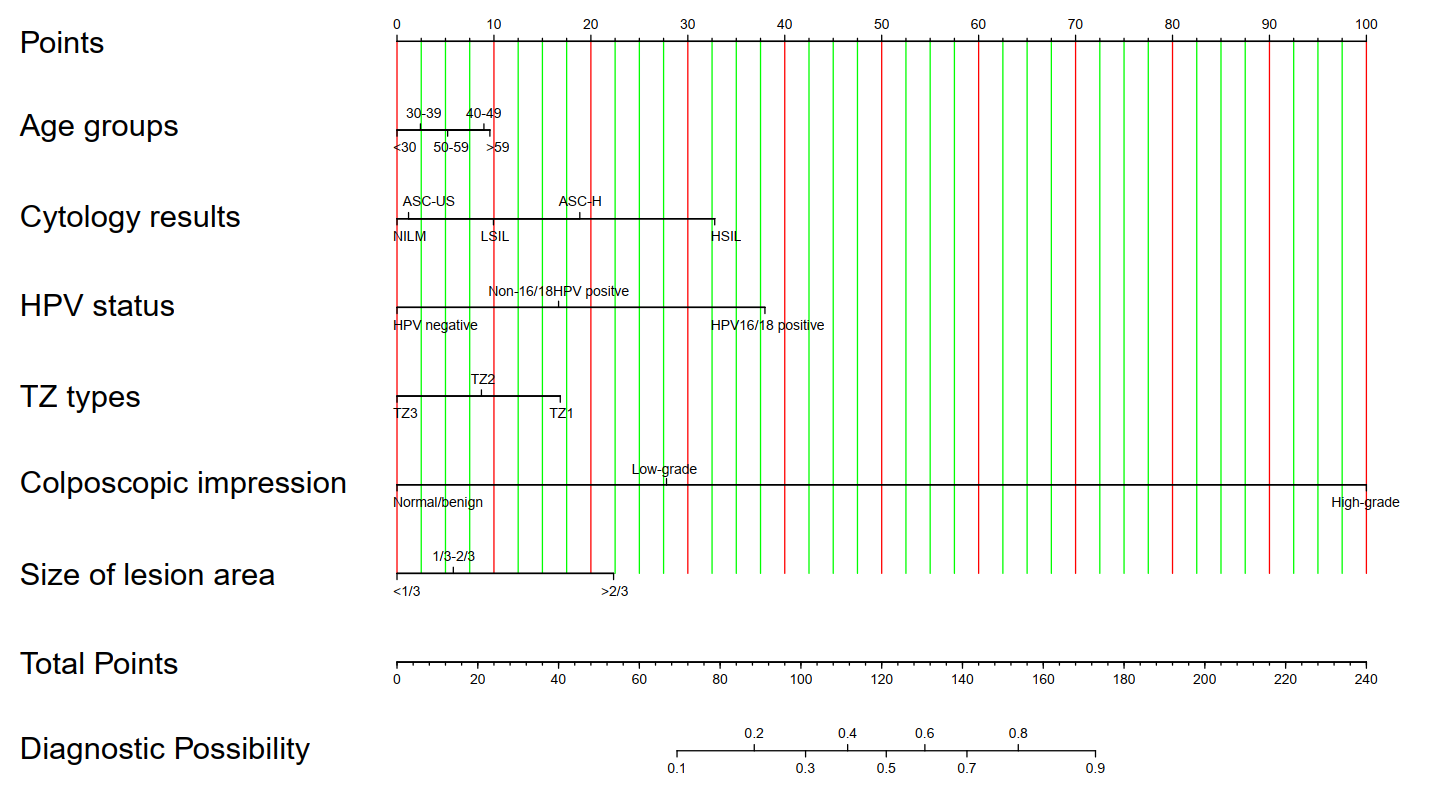
Figure S2**. Nomogram of HSIL+ risk prediction.

| **Table S5.** Points per unit of each variable in the Nomogram. | |
| --- | --- |
| Variables | Points |
| Age groups | |
| <30 | 0 |
| 30-39 | 2 |
| 40-49 | 9 |
| 50-59 | 5 |
| >60 | 10 |
| Cytology results | |
| NILM | 0 |
| ASC-US | 1 |
| LSIL | 10 |
| ASC-H | 19 |
| HSIL | 33 |
| HPV status | |
| HPV negative | 0 |
| Non-16/18 hr-HPV | 17 |
| HPV16/18 positive | 38 |
| TZ types |  |
| TZ1 | 17 |
| TZ2 | 9 |
| TZ3 | 0 |
| Colposcopic impression |  |
| Normal/benign | 0 |
| Low-grade | 28 |
| High-grade | 100 |
| Size of lesion area |  |
| <1/3 | 0 |
| 1/3-2/3 | 6 |
| >2/3 | 22 |

As a practical example, we assessed a patient’s risk, as follows: The individual, labelled #1, was 35 years old (2 points) with cytology results of NILM (0 points), non-16/18 HPV positive (17 points), TZ3 (0 points), colposcopic impression of normal/benign (0 points), and size of lesion area of 1/3-2/3 (6 points), and individual 2 was 51 years old (5 points) with cytology results of ASC-H (19 points), HPV16/18 positive (38 points), TZ2 (9 points), colposcopic impression of high-grade (100 points), and size of lesion area of 1/3-2/3 (0 points). The total score for various predictors was approximately 25 points and 171 points. The corresponding absolute predicted risk of developing HSIL+ before the time of colposcopy-guided biopsy was around 0.004 and 0.890 for individuals 1 and 2, respectively.
